# Elevated cortisol concentration in preterm sheep fetuses impacts heart development

**DOI:** 10.1113/EP092506

**Published:** 2025-04-28

**Authors:** Reza Amanollahi, Stacey L. Holman, Melanie R. Bertossa, Ashley S. Meakin, Vicki L. Clifton, Kent L. Thornburg, I. Caroline McMillen, Michael D. Wiese, Mitchell C. Lock, Janna L. Morrison

**Affiliations:** ^1^ Early Origins of Adult Health Research Group, Health and Biomedical Innovation; UniSA: Clinical and Health Sciences University of South Australia Adelaide South Australia Australia; ^2^ Pregnancy and Development Group, Mater Research Institute University of Queensland South Brisbane Queensland Australia; ^3^ Department of Medicine, Center for Developmental Health, Knight Cardiovascular Institute, Bob and Charlee Moore Institute of Nutrition and Wellness Oregon Health & Science University Portland Oregon USA; ^4^ Centre for Pharmaceutical Innovation Clinical & Health Sciences University of South Australia Adelaide South Australia Australia

**Keywords:** cardiovascular diseases, cortisol, heart development, mid‐late gestation, preterm fetus

## Abstract

The prepartum rise in cortisol promotes cardiac development and maturation. Here, we investigated the impact of elevated circulating cortisol during mid‐late gestation on cardiac growth and metabolism in fetal sheep. Saline or cortisol (2–3 mg in 4.4 mL/24 h) was infused into the fetal jugular vein from 109 to 116 days gestation (dG, term = 150 dG), and fetal heart tissue was collected at 116 dG. Glucocorticoid concentrations, gene and protein expression were measured in fetal left ventricle (LV) tissue. Intrafetal cortisol infusion increased cardiac cortisol concentration but downregulated the protein abundance of glucocorticoid receptor (GR) isoforms (GRα‐A, GR‐P, GR‐A, GRα‐D2 and GRα‐D3). The gene and protein expression of markers of cardiac hyperplastic growth (*IGF1*, IGF‐1R, *TGFβ* and *AGT*) were downregulated, while a protein marker of DNA replication (proliferating cell nuclear antigen) was upregulated by cortisol infusion. Cardiac protein and/or gene expression of complex I of the electron transport chain, *SOD2*, GLUT‐4 (gene and protein), and phosphorylated IRS‐1, were upregulated in response to elevated fetal cortisol concentration. Intrafetal cortisol infusion downregulated gene expression of *PDK4*, which mediates the metabolic switch from glucose to fatty acid metabolism. Cardiac expression of molecular markers involved in cardiovascular protection (SIRT‐1, *HO1*, *LAMP1* and *SK1*) were also downregulated in the cortisol group. In conclusion, these findings suggest that chronic cortisol exposure in preterm fetuses alters heart development, promoting cardiac maturation and potentially increasing the risk of cardiovascular disease later in life if these changes persist into adulthood.

## Abstract (highlights)

- **What is the central question of this study?**
  Can elevated fetal cortisol concentration at earlier gestational age impact heart development?
- **What is the main finding and its importance?**
  Excess cortisol exposure in preterm fetuses impacts heart development and may increase the risk of cardiovascular disease later in life if changes persist into adulthood.

## 1 INTRODUCTION

Cardiovascular disease (CVD) remains the number one cause of death worldwide, and several risk factors such as age, pre‐existing conditions and lifestyle contribute to its disease burden (Ciumărnean et al., 2022; Townsend et al., 2022; Tsao et al., 2022). However, there is much to gain by exploring the cause of CVD from a different angle: the impact of endogenous and exogenous factors in fetal life when cardiac structure and function are developing, an established discipline known as developmental programming (Barker et al., 1989; McMillen & Robinson, 2005; Miranda et al., 2017; Zhu et al., 2019). During fetal heart development, there are complex molecular changes that facilitate the transition from fetal to adult life. These processes result in mature cardiomyocytes that beat for a lifetime, with limited capacity for cell regeneration or repair after birth in large mammals, including sheep and humans (Bergmann et al., 2009; Dimasi et al., 2023; Guo & Pu, 2020; Jonker et al., 2010; Lock et al., 2018). Therefore, stressors that create a suboptimal environment for a fetus during this maturational process, including reduced nutrients, hypoxemia, and changes in circulating hormones, can result in poor long‐term cardiac health (Darby, Varcoe, et al., 2020; Louey & Thornburg, 2005; Morrison, 2008).

Glucocorticoids such as cortisol, the primary glucocorticoid in humans and sheep, and corticosterone, the equivalent of cortisol in mice and rats, are steroid hormones produced by the adrenal cortex in response to physical, psychological and environmental stressors (Cruz‐Topete et al., 2020; Rog‐Zielinska et al., 2014). The prepartum surge in fetal plasma cortisol concentration begins at ∼134 days of gestation (dG; term = 150 dG) in sheep, and at ∼30 weeks (term, 40 weeks) in humans, with a peak before birth that triggers maturation of organ systems, including the heart, via glucocorticoid receptor (GR)‐mediated signalling pathways (Dimasi et al., 2023; Fencl et al., 1980; Hillman et al., 2012; Phillips et al., 1996). Several protein isoforms of the GR have been identified in the human and sheep placenta and lung (Clifton et al., 2019; Orgeig et al., 2015; Saif et al., 2014) that can modulate canonical GR‐mediated signalling pathways. However, the expression of GR isoforms, their response to circulating cortisol, and how this influences cardiac maturation in the preterm fetus are unknown. Filling this knowledge gap is important as the normal prepartum surge in cortisol also causes an increase in thyroid hormone concentration, and both cortisol and thyroid hormone play important roles in cardiac maturation (Chattergoon et al., 2007, 2012; Naqvi et al., 2014; Thornburg et al., 2011). However, pregnancy complications can lead to a premature increase in cortisol concentrations earlier in gestation that impacts the developing fetus and its cardiovascular outcomes. These include maternal stress (Eberle et al., 2021), maternal health conditions such as gestational diabetes mellitus (Tien Nguyen et al., 2023), preeclampsia (Aufdenblatten et al., 2009), obesity (Zambrano & Nathanielsz, 2013), and placental insufficiency (Gagnon, 2003). For example, placental insufficiency leading to fetal growth restriction is associated with elevated plasma cortisol concentrations as well as a reduction in cardiomyocyte proliferation, with larger cardiomyocytes in late gestation and fewer cardiomyocytes both in the neonatal and adolescent period (Botting et al., 2018, 2014; Louey & Thornburg, 2005; Morrison et al., 2007). Hence, pregnancy complications and adverse intrauterine environment can modulate GR‐mediated signalling pathways, thereby influencing fetal cardiac signalling mechanisms.

Around the time of birth, fetal cardiac cells shift from glycolysis to oxidative phosphorylation (OXPHOS), which generates ATP more efficiently to deal with the increased cardiac energy demand of postnatal life (Lopaschuk & Jaswal, 2010). This switch to OXPHOS is associated with a shift away from predominantly glucose and lactate to free fatty acids as the major source of ATP production (Dimasi et al., 2023; Fisher et al., 1982; Lopaschuk & Jaswal, 2010). In fetal life, glucose is a metabolic substrate for the fetal heart more than fatty acids (Shao & Tian, 2015), with glucose transporters 1 and 4 (GLUT‐1, and GLUT‐4) being the major GLUTs responsible for cardiac glucose uptake (Shao & Tian, 2015; Szablewski, 2017). Previous studies have highlighted the role of elevated cortisol in altering fetal metabolism (Fowden et al., 1996; Jellyman et al., 2012; Vaughan et al., 2018). Specifically, elevated fetal exposure to cortisol in late gestation in the sheep fetus impacts fetoplacental metabolism with a reduction in umbilical glucose uptake (Vaughan et al., 2018), increased glucose storage in several fetal tissues (Fowden et al., 1996) and increased GLUT‐4 abundance in skeletal muscle (Jellyman et al., 2012). However, the impact of elevated cortisol on the cardiometabolic profile of the fetal heart remains to be fully elucidated.

Animal models have been used to study heart development and disease. Mice and rats are often utilized for their short gestation; however, unlike humans, mice and rats are born relatively immature with a heart containing proliferative cardiomyocytes (Botting et al., 2012; Liu et al., 2010; Soonpaa et al., 1997). Cardiac maturation in large animals such as sheep is similar to humans, where the contractile units of the heart begin the transition from proliferative to quiescent before birth (Burrell et al., 2003; Lock et al., 2018; Morrison et al., 2018), coinciding with the prepartum rise in cortisol. Previous studies of fetal cortisol infusion resulted in supraphysiological increases in plasma cortisol concentration and led to effects on heart weight and markers of cardiac growth through proliferation and hypertrophy (Giraud et al., 2006; Lumbers et al., 2005). Cortisol infusion into preterm fetuses either inhibited cardiomyocyte proliferation (Rudolph et al., 1999) or had no effect on the rate of terminal differentiation and number of cardiomyocytes (Lumbers et al., 2005). In contrast, others found an increase in fetal heart mass due to cardiomyocyte proliferation rather than hypertrophy (Giraud et al., 2006). Despite these conflicting findings, no studies have specifically explored the effects of cortisol on markers of cardiac protection, metabolism and the expression of GR isoforms. Therefore, we hypothesized that intrafetal cortisol infusion in a preterm sheep fetus would increase cortisol in cardiac tissue, decrease GR protein expression, and promote an early switch from hyperplastic to hypertrophic growth.

## 2 METHODS

### 2.1 Ethics and animal husbandry

All procedures were approved by the University of Adelaide Animal Ethics Committee (Ethics no. M/70/95) and comply with the Australian code of practice for the care and use of animals for scientific purposes. All investigators understood and followed the ethical principles outlined in Grundy (2015) and the principles of the 3Rs, specifically the reduction of the use of animals in research (Russell & Burch, 1959). The ARRIVE guidelines were also understood and followed where applicable (Percie du Sert et al., 2020). Sixteen pregnant Border–Leicester×Merino and Merino ewes (carrying singletons) were housed in individual pens in animal rooms (located in the University of Adelaide Medical School), with a 12:12 h light/dark cycle, and received 100% of metabolizable energy requirements once daily with water ad libitum. All researchers were blinded to the treatment groups while assessing the experimental protocols.

### 2.2 Fetal surgery

At 103 dG (term = 150 dG), pregnant ewes underwent surgery with general anesthesia induced with an intravenous injection of sodium thiopentone (1.25 g, Pentothal; Rhone Merieux, Pinkenba, Qld, Australia) and maintained with 2.5–4% halothane inhalation anaesthetic (Fluothane; ICI, Melbourne, Vic, Australia) in oxygen. Vascular catheters were inserted into the maternal jugular vein, fetal jugular vein, fetal carotid artery and amniotic cavity as described previously (McGillick et al., 2013; Ross et al., 2000). Fetal catheters were exteriorized through an incision in the ewe's right flank. The ewes received an intramuscular injection of antibiotics [3.5 mL of procaine penicillin 150 mg/mL and benzathine penicillin 112.5 mg/mL (Norocillin, Norbrook Laboratories Ltd, Newry, UK) and 2 mL of 125 mg/mL dihydrostreptomycin in sterile saline (Sigma‐Aldrich, St Louis, MO, USA)], as well as a post‐operative analgesic (20 µg/kg, Xylazil; Troy Laboratories, Glendenning, NSW, Australia) at surgery and for 3 days post‐surgery. The fetuses also received antibiotics at surgery and intra‐amniotically for 4 days post‐surgery (500 mg; sodium ampicillin; Commonwealth Serum Laboratories, Melbourne, Vic, Australia). The ewes were allowed 6 days to recover from surgery prior to the experimental protocol. To monitor fetal health, arterial blood samples (∼1 mL) were collected from fetuses daily for measuring $P_{aO_{2}}$, $P_{\mathrm{aC}O_{2}}$, pH, oxygen saturation ($S_{aO_{2}}$) and haemoglobin (Hb) using an ABL 550 analyser (Radiometer Pacific Pty Ltd, Lane Cove West, NSW, Australia) and temperature corrected to 39°C (Adams et al., 1999; McGillick et al., 2013).

### 2.3 Infusion protocol and cortisol assay

Ewes were randomly assigned to either the Saline group (infusion of normal saline (4.4 mL/24 h); *n* = 8, 3 male, 5 female) or the Cortisol group (infusion of cortisol (hydrocortisone succinate, Solucortef, Upjohn Pharmaceuticals Ltd, Hastings, MI, USA, 2–3 mg in 4.4 mL/24 h); *n* = 8, 5 male, 3 female) into the fetal jugular vein from 109 to 116 dG (Adams et al., 1999; Williams et al., 2004). Total cortisol concentrations were measured in fetal plasma by radioimmunoassay (RIA) using an Orion Diagnostica kit (Orion Diagnostica, Turku, Finland), as previously described (Adams et al., 1999). The impact of Saline versus Cortisol infusion in this cohort on the adrenal gland and lung development has been previously published (Adams et al., 1999; McGillick et al., 2013).

### 2.4 Post‐mortem and tissue collection

At 116 dG, ewes were humanely killed with an intravenous overdose of sodium pentobarbitone (150 mg/kg, Virbac Pty Ltd, Peakhurst, NSW, Australia), and fetuses were delivered by hysterectomy, weighed and body measurements were taken. The fetal hearts were removed, weighed, and a sample of the left ventricle (LV) was snap‐frozen in liquid nitrogen, and stored at −80°C, as well as fixed in 4% paraformaldehyde for subsequent molecular and histological analysis.

### 2.5 Quantification of fetal cardiac mRNA expression

RNA was extracted using the RNeasy Mini Kit (Qiagen, Hombrechtikon, Switzerland) following the manufacturer's instructions (TRIzol reagent, Thermo Fisher Scientific, Waltham, MA, USA; tissue homogenizer, TissueLyser LT, Qiagen, Hilden, Germany), and cDNA synthesized from a subset of fetal LV tissues (∼50 mg; Saline, *n* = 7; Cortisol, *n* = 8) as previously described (Lie, Morrison, et al., 2014; McGillick et al., 2013; Soo et al., 2012), and following the MIQE guidelines (Bustin et al., 2009). Briefly, the total RNA was quantified using spectrophotometric measurements at 260 and 280 nm in a NanoDrop Lite Spectrophotometer (Thermo Fisher Scientific), followed by running on the gel to check for sufficient integrity. For cDNA synthesis, Superscript III (Thermo Fisher Scientific) was utilized with 1 µg of total RNA, random hexamers, dNTP, and dithiothreitol in a final volume of 20 µL, following the manufacturer's guidelines in a MJ Mini personal thermocycler (Bio‐Rad Laboratories, Hercules, CA, USA). Controls were included to detect reagent and genomic DNA contamination, using either no RNA transcript or no Superscript III, respectively. To ensure RNA samples contained no double‐stranded DNA, all samples were run on a no‐amplification control (NAC) plate. The geNorm component of qbase^plus^ software (version 2.0, Biogazelle, Ghent, Belgium) was employed to identify the most stable reference genes across all samples from a panel of eight candidate housekeeping genes (Vandesompele et al., 2002), and the minimum number of reference genes required to calculate a stable normalization factor, as previously described (Lie, Morrison, et al., 2014; McGillick et al., 2013; Soo et al., 2012). The expression of target genes regulating cardiac hyperplastic growth (*IGF1*, *IGF2*, *IGF1R*, *IGF2R*, *IGFBP2*, *RPS6KB1*, *ANP*, *TGFβ*, *PCNA*, *P21*, *P27*, *CYCLIN D1*, *GATA4*, *GATA6*, *CDC2*, *CHEK1*, *C‐KIT*, *CD31*), angiogenesis (*VEGF*, *ANGIOPOIETIN‐1*, *ANGIOPOIETIN‐2*, *PAI‐1*), the renin–angiotensin system (*AGT*, *RENIN*, *AT1R*, *AT2R*, *ACE1*, *ACE2*), oxidative stress (*SOD1*, *SOD2*, *SOD3*, *CAT*, *eNOS*, *iNOS*), apoptosis (*BAX*, *BCL2*, *c‐MYC*, *BIRC5*), autophagy (*BECLIN1*, *ATG1*), glucocorticoid signalling (*GR*, *MR*, *11βHSD1*), glucose uptake (*GLUT1*, *GLUT4*), fatty acid metabolism (*PPARγ*, *PDK4*) and cardiovascular protection (*HO1*, *LAMP1*, *SK1*) were measured by qRT‐PCR (KiCqStart SYBR Green qPCR ReadyMix Low Rox (Sigma‐Aldrich, St Louis, MO, USA) in a final volume of 6 µL) using previously optimized primers designed for sheep (Botting et al., 2014; Darby et al., 2019; Orgeig et al., 2015; Wang et al., 2012, 2013, Wang, Brooks, et al., 2015) on a ViiA7 Fast Real‐time PCR system (Thermo Fisher Scientific). The abundance of each transcript relative to the abundance of selected stable reference genes (peptidylprolyl isomerase A, *PPIA*; hypoxanthine phosphoribosyltransferase 1, *HPRT*) was calculated using DataAssist 3.0 analysis software (Thermo Fisher Scientific) and expressed as mRNA mean normalized expression (MNE) ± SD, as previously described (Lie, Morrison, et al., 2014; McGillick et al., 2013; Soo et al., 2012).

### 2.6 Quantification of fetal cardiac protein expression

Fetal LV tissue (∼100 mg; Saline *n* = 8, Cortisol *n* = 8) was initially pulverized and then sonicated (John Morris Scientific, Adelaide, SA, Australia) in a lysis buffer containing Tris–HCl (50 mM), NaCl (150 mM), NP‐40 (1%), sodium orthovanadate (1 mM), sodium fluoride (30 mM), sodium pyrophosphate (10 mM), EDTA (10 mM), and protease inhibitor (1 tablet/20 mL buffer; complete Mini, Roche, Basel, Switzerland). Samples were centrifuged at 14,300 *g* at 4°C for 14 min (Eppendorf Centrifuge 5415, Crown Scientific, Vic, Australia). A Pierce Micro BCA Protein Assay Kit (Thermo Fisher Scientific) was used to determine the protein concentration of each sample. BSA (2 mg/mL stock solution) was used for the standard curve. Extracted protein samples (5 mg/mL) were loaded onto SDS‐PAGE gels (12%) and stained with Coomassie blue to confirm a consistent concentration of protein for all diluted samples. An equal volume (15 µL) of each sample was resolved by SDS‐PAGE (10%–15%). The resolved proteins were then transferred onto a nitrocellulose membrane (Hybond ECL, GE Healthcare, Mascot, NSW, Australia), which were subsequently stained with Ponceau S solution (0.1% (w/v) in 5% acetic acid, Sigma‐Aldrich), followed by imaging with ImageQuant LAS4000 (GE Healthcare, Melbourne, VIC, Australia) to confirm adequate protein transfer. The membrane was then washed with Tris‐buffered saline (TBS, 3 × 5 min), and blocked in 5% BSA in TBS with 1% Tween (TBS‐T) for 1 h at room temperature. The membranes underwent washes in TBS‐T (3 × 5 min) and were cut according to the size of the proteins prior to incubation at 4°C overnight with their respective primary antibodies. Target proteins were selected based on previous studies (Botting et al., 2018, 2014; Darby, Sorvina, et al., 2020; Lie, Hui, et al., 2014; Saif et al., 2014; Wang, Tosh, et al., 2015) indicating their critical roles in fetal cardiometabolic pathways, as summarized in Supporting information, Table S1. All antibodies were diluted in 5% BSA in TBS‐T. Membranes were again washed in TBS‐T (3 × 5 min) before being incubated with the appropriate horseradish peroxidase‐labelled secondary IgG antibody (1:2000, anti‐mouse, cat. no. 7076, anti‐rabbit, cat. no. 7054, Cell Signaling Technology, Danvers, MA, USA) for 1 h at room temperature. Enhanced chemiluminescence using SuperSignal West Pico Chemiluminescent Substrate (Thermo Fisher Scientific) was used to detect bands on the blots. The western blot was imaged using ImageQuant LAS 4000 (GE Healthcare, Melbourne, VIC, Australia), and the protein abundance was quantified by densitometry using ImageQuant TL 8.1 software (GE Healthcare). The abundance of target proteins was then normalized to a reference protein, Vinculin (1:2000, cat. no. 18799S, Cell Signaling Technology).

### 2.7 Quantification of fetal cardiac concentration of glucocorticoid and thyroid hormones

Tissue hormone concentrations were determined by liquid chromatography (LC; Shimadzu Nexera XR, Shimadzu, Japan) coupled to a SCIEX 6500 Triple‐Quad system (MS/MS; SCIEX, US) using an adapted protocol (Dimasi, Darby, Cho, et al., 2024; McBride et al., 2021). Initially, a subset of LV tissue (Saline, *n* = 7; Cortisol, *n* = 8) was homogenized in 500 µL 0.9% NaCl at 50 Hz for 2 min and then centrifuged at 12,000 *g* for 10 min at 4°C. Supernatant (100 µL) was added to 300 µL acetonitrile containing 50 ng/ml internal standard (cortisol‐9,11,12,12‐d4; Toronto Research Chemicals, Toronto, Canada), vortexed for 1 min and then centrifuged at 12,000 *g* for 10 min. Supernatant was transferred to a fresh Eppendorf tube and the remaining pellet was resuspended in 300 µL ethyl acetate, vortexed for 1 min, and then centrifuged at 12,000 *g* for 10 min. Supernatant was added to the acetonitrile, mixed by inversion, and then evaporated to dryness using the GeneVac EZ‐2 Evaporating System (GeneVac, Ipswich, UK). Dried samples were reconstituted in 50% methanol and then injected onto an ACQUITY UPLC BEH C18 Column (130 Å, 1.7 µm, 2.1  × 100 mm; Waters Corp., Milford, MA, USA). Mobile phases were 0.1% formic acid in water (A) and 0.1% formic acid in acetonitrile (B). The flow rate was 0.3 mL/min and mobile phase B was initially 10% and increased linearly to 90% over 10 min and then held at 90% for 2 min, after which it was returned to 10% for 3 min prior to injection of the next sample. Hormone concentrations were calculated via integration with a standard curve that ranged from 0.05 to 100 ng/ml. Conditions for detection of analytes are as previously described (Bertossa et al., 2024; Lock et al., 2023; McBride et al., 2021).

### 2.8 Quantification of fetal cardiac enzymatic activity

A lactate dehydrogenase (LDH) assay kit (cat. no. ab102526, Abcam, Cambridge, UK) and citrate synthase (CS) assay kit (cat. no. CS0720, Sigma‐Aldrich) were used to quantify the enzymatic activities of LDH and CS, respectively, in fetal LV tissue (∼100 mg; Saline, *n* = 8; Cortisol, *n* = 8). The assays were performed according to manufacturer protocol and previously described in detail (Dimasi, Darby, Holman et al., 2024).

### 2.9 Quantification of fetal cardiac glycogen and collagen staining

Paraformaldehyde‐fixed paraffin‐embedded blocks from a subset of fetal LV (Saline, *n* = 5, Cortisol, *n* = 5) were sectioned at 5 µm (rotary microtome) onto Superfrost slides (VWR International, Randor, PA, USA). Periodic acid–Schiff (PAS; glycogen) and Masson's trichrome (collagen) staining were performed by the University of Adelaide Histology Services. The slides were then scanned at ×40 magnification using a NanoZoomer‐XR (Hamamatsu, Hamamatsu City, Japan) to generate whole‐slide images. PAS slides were analysed by Fiji/ImageJ software (version 1.54f, NIH, Bethesda, MD, USA) using the colour saturation threshold tool at ×20 magnification (five frames 1 mm apart). Masson's trichrome slides were analysed in the VIS software suite version (Visiopharm 2020.08, Horsholm, Denmark) using custom threshold application at ×10 magnification (whole slide) as previously described (Lock et al., 2019). Correct quantification of the staining was confirmed by visual examination by a trained individual who was blinded to the treatment groups.

### 2.10 Quantification of fetal cardiac Ki67 by immunohistochemistry

Paraformaldehyde‐fixed paraffin‐embedded blocks from a subset of fetal LV (Saline, *n* = 5, Cortisol, *n* = 5) were sectioned at 5 µm (rotary microtome) onto Superfrost slides. Slides were baked at 60°C for 1 h followed by deparaffinization and rehydration. After rehydration, endogenous peroxide activity was blocked with 3% hydrogen peroxide (Sigma‐Aldrich), followed by heat‐induced antigen retrieval in citrate buffer (pH 6.0). Slides were incubated overnight with the primary antibody (Ki67, 1:200, cat. no. M7240, Agilent Technologies, Santa Clara, CA, USA) at 4°C following incubation with non‐immune serum (serum blocking solution; Thermo Fisher Scientific) to prevent non‐specific binding. Negative control slides with the primary antibody omitted were used to demonstrate no non‐specific binding of the secondary antibody or reagent contamination. In addition, negative control slides where incubation with primary antibody was substituted for mouse serum (Sigma‐Aldrich), and then incubated overnight at 4°C. A Metal Enhanced DAB Substrate Kit (cat. no. 34065, Thermo Fisher Scientific) was used for visualization of positive cells. All sections were counterstained with Mayer's haematoxylin (Sigma‐Aldrich). Each final antibody concentration was optimized within the immunohistochemistry (IHC) protocol (as above) by manipulating a range of test conditions, as previously described (Lock et al., 2019, 2015). The stained slides were scanned using a NanoZoomer‐XR (Hamamatsu), and then analysed (whole tissue) by QuPath software (version 0.4.4) to determine the percentage of Ki67 positive cells in fetal LV tissue. Correct quantification of the staining was confirmed by visual examination by a trained individual who was blinded to the treatment groups.

### 2.11 Statistical analysis

All statistical analyses were performed using GraphPad Prism 10 (GraphPad Software, Boston, MA, USA). Some samples were not included in analysis due to missing animal records (fetal parameters), systematic or technical errors (mRNA expression and hormone assay), or missing fixed tissue samples (histology). Outliers were removed from each group using the Grubbs's method (α = 0.05), when applicable. In the western blot data, any non‐quantifiable bands (due to artifacts affecting the bands) were excluded from the analysis and are indicated on the blots with an X. An additional control group was initially included on the same blots to compare the saline and cortisol groups with the older gestational age control group. However, after analysing the data, we chose to exclude the older fetuses from this study to ensure a more concise and coherent message throughout the paper. The number of samples used for each analysis is listed within table and figure legends. Data were assessed for normality (Shapiro–Wilk test). Analysis of outcomes in Saline versus Cortisol animals was performed using an unpaired Student's *t*‐test if data were normally distributed, otherwise a Mann–Whitney test was used. To assess the relationship between the two measures, simple linear regression was used. The effect of sex was not evaluated due to an insufficient number of samples; however, sex is indicated in figures by different symbols. Data in figures and tables are presented as means ± SD. A *P*‐value of < 0.05 was considered statistically significant.

## 3 RESULTS

### 3.1 Intrafetal cortisol infusion did not impact fetal body and heart weight or fetal blood gas measures

During the 7‐day cortisol infusion, fetal blood gas and metabolites ($P_{aO_{2}}$, $P_{\mathrm{aC}O_{2}}$, pH and haemoglobin) were not different from either baseline (the day before infusion) or Saline fetuses as previously described (McGillick et al., 2013). Measures of fetal body and heart weight (absolute and relative) were not different between Saline and Cortisol fetuses (Table 1).

**TABLE 1:** Intrafetal cortisol infusion did not impact fetal heart or body growth.

Fetal parameter	Saline (*n* = 8)	Cortisol (*n* = 8)	*P*
Body weight (kg)	2.11 ± 0.36 (*n* = 7)	2.14 ± 0.32	0.8359
Crown‐rump length (cm)	45.2 ± 2.73 (*n* = 7)	43.2 ± 1.21 (*n* = 7)	0.1028
Heart weight (g)	16.89 ± 4.52	16.79 ± 3.22	0.9606
Heart weight: body weight (g/kg)	8.07 ± 1.62 (*n* = 7)	7.79 ± 0.78	0.6684
LV weight (g)	9.21 ± 3.12 (*n* = 7)	8.56 ± 2.42	0.6564
LV weight: body weight (g/kg)	4.26 ± 1.37 (*n* = 6)	3.94 ± 0.75	0.5847
RV weight (g)	5.17 ± 0.9 (*n* = 7)	4.38 ± 0.86 (*n* = 7)	0.1213
RV weight: body weight (g/kg)	2.38 ± 0.44 (*n* = 6)	1.99 ± 0.34 (*n* = 7)	0.1056

*Note*: Data expressed as means ± SD. Data were analysed by unpaired *t*‐test. *P* < 0.05 was considered statistically significant. Animal numbers used within each subset of the study are shown in brackets. Data were excluded due to missing records. Abbreviations: LV, left ventricle; RV, right ventricle.

### 3.2 Intrafetal cortisol infusion increased cortisol concentrations in the fetal heart, but decreased markers of glucocorticoid signalling

**FIGURE 1:**
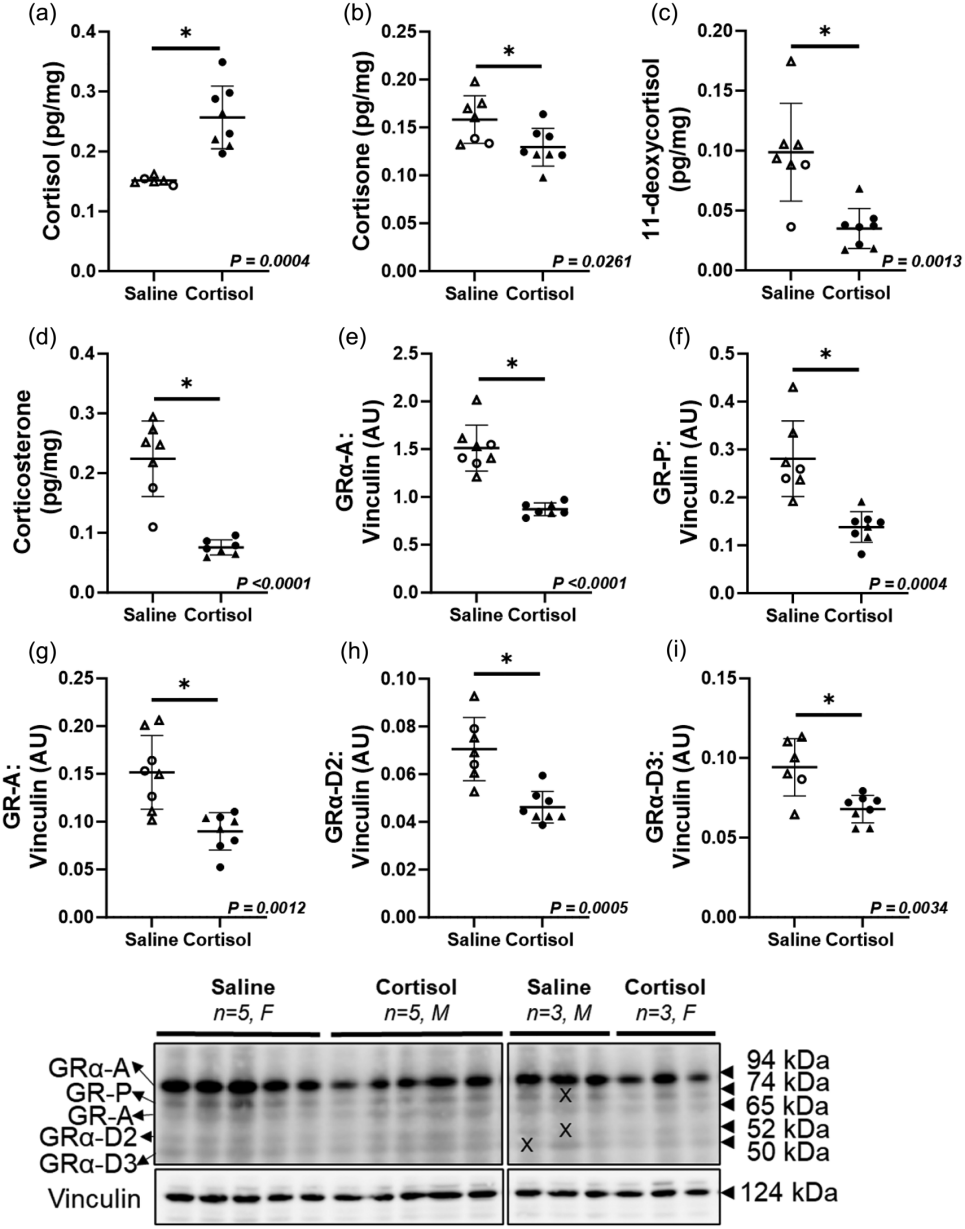
Intrafetal cortisol infusion increased fetal cardiac cortisol concentration, while decreasing markers of glucocorticoid signalling. (a–d) There was an increase in the cortisol concentration within the fetal heart (a), while the cortisol metabolites including cortisone (b), 11‐deoxycortisol (c) and corticosterone (d) were decreased by intrafetal cortisol infusion. (e–i) The protein expression of GR isoforms including GRα‐A (e), GR‐P (f), GR‐A (g), GRα‐D2 (h) and GRα‐D3 (i) were decreased by cortisol infusion. Males (M), circles; females (F), triangles. Saline, LV tissue from saline‐infused fetuses, open symbols (hormones: 2M, 5F; protein: 3M, 5F); Cortisol, LV tissue from cortisol‐infused fetuses, filled symbols (hormones: 5M, 3F; protein: 5M, 3F). LC‐MS/MS and western blot were run in one sample per animal. Data were excluded due to technical error for hormone concentration. One outlier was excluded per group using the Grubbs method (α = 0.05), when applicable. Data expressed as means ± SD and were analysed by the unpaired *t*‐test. *P *< 0.05 was considered significant. X indicates data excluded from analysis (due to artifacts affecting the bands). AU, arbitrary unit; GR, glucocorticoid receptor; LV, left ventricle; MNE, mean normalized expression.

Cortisol infusion increased tissue cortisol concentration within the fetal heart (*P *= 0.0004; Figure 1a), while other glucocorticoids (cortisone, *P *= 0.0260; 11‐deoxycortisol, *P *= 0.0013; and corticosterone, *P *< 0.0001) were lower in the Cortisol compared to the Saline group (Figure 1b–d). Cortisol infusion also increased fetal plasma cortisol concentrations (*P *= 0.0005) when compared with saline‐infused fetuses (Saline, 0.616 ± 0.036 ng/mL; Cortisol, 15.62 ± 1.49 ng/mL) as previously published using RIA (Adams et al., 1999). The cardiac protein expression of five GR isoforms (GRα‐A, *P *≤ 0.0001; GR‐P, *P *= 0.0004; GR‐A, *P *= 0.0012; GRα‐D2, *P *= 0.0005; and GRα‐D3, *P *= 0.0034) was decreased by intrafetal cortisol infusion (Figure 1e–i). Cardiac hormone concentration of thyroid hormones (triiodothyronine, T_3_; thyroxine, T_4_), and mRNA expression of *GR* (*NR3C1*), *MR* (*NR3C2*) and *11βHSD1* was not different between Saline and Cortisol groups (Table 2).

**TABLE 2:** Effect of intrafetal cortisol infusion on fetal cardiac hormones and expression of genes or proteins involved in glucocorticoid signalling, cardiac hyperplastic growth, OXPHOS, apoptosis, autophagy, angiogenesis, cardiac contractility, renin–angiotensin system, oxidative stress and glucose/fatty acid metabolism.

	Saline (*n* = 8)	Cortisol (*n* = 7)	*P*
Hormones in cardiac tissue			
T_3_ (pg/mg)	0.0062 ± 0.0006 (*n* = 7)	0.0079 ± 0.0022 (*n* = 8)	0.0753
T_4_ (pg/mg)	0.2823 ± 0.0142 (*n* = 7)	0.3241 ± 0.0752 (*n* = 8)	0.6620^a^
Progesterone (pg/mg)	0.0333 ± 0.0138 (*n* = 7)	0.0343 ± 0.0271 (*n* = 8)	0.5358^a^
Glucocorticoid signalling			
*GR* (MNE)	0.9407 ± 0.2181	0.8412 ± 0.1323	0.3145
*MR* (MNE)	0.1405 ± 0.0439 (*n* = 7)	0.1318 ± 0.0315 (*n* = 6)	0.6942
*11βHSD1* (MNE)	0.0249 ± 0.0270 (*n* = 7)	0.0127 ± 0.0043	>0.9999^a^
Cardiac hyperplastic growth			
*IGFBP2* (MNE)	0.2019 ± 0.0936	0.1508 ± 0.0421	0.2072
*IGF1R* (MNE)	0.7362 ± 0.2439	0.6684 ± 0.2018	0.5711
*IGF2R* (MNE)	2.073 ± 0.3073	2.066 ± 0.4110	0.9694
*RPS6KB1* (MNE)	0.1628 ± 0.0276 (*n* = 6)	0.1703 ± 0.0231 (*n* = 6)	0.6240
*ANP* (MNE)	1.945 ± 4.226	0.3976 ± 0.3198	0.5350^a^
*PCNA* (MNE)	0.1810 ± 0.0209	0.1900 ± 0.0513	0.6568
*P21* (MNE)	0.02468 ± 0.0255 (*n* = 7)	0.01311 ± 0.0064 (*n* = 6)	0.7308^a^
*P27* (MNE)	0.4568 ± 0.1162	0.3982 ± 0.0447	0.5358^a^
*CYCLIN D1* (MNE)	0.06554 ± 0.0168	0.06498 ± 0.0178	0.9514
*GATA4* (MNE)	0.3428 ± 0.1129 (*n* = 6)	0.3508 ± 0.0649	0.8769
*GATA6* (MNE)	2.038 ± 0.3044	1.779 ± 0.1357	0.0819
*CDC2* (MNE)	0.2523 ± 0.0654	0.2753 ± 0.0798	0.5500
*CHEK1* (MNE)	0.0665 ± 0.0099	0.0622 ± 0.0193	0.5945
*C‐KIT* (MNE)	0.0888 ± 0.0165	0.0817 ± 0.0201	0.4829
*CD31* (MNE)	0.9589 ± 0.1378	0.8723 ± 0.1712	0.2973
p‐mTOR:m‐TOR ratio (AU)	0.2788 ± 0.1186	0.2662 ± 0.1018	0.8309
p‐P70 S6K:P70 S6K ratio (AU)	0.6291 ± 0.0922	0.5816 ± 0.1009	0.3427
Apoptosis			
*BAX* (MNE)	0.1117 ± 0.0080 (*n* = 6)	0.1092 ± 0.0156	0.7500
*BCL2* (MNE)	0.0117 ± 0.0021 (*n* = 6)	0.0097 ± 0.0019	0.1118
*c‐MYC* (MNE)	0.1326 ± 0.0339 (*n* = 6)	0.1339 ± 0.0487	0.9576
*BIRC5* (MNE)	0.1571 ± 0.0601	0.1661 ± 0.0625	0.7810
p‐FOXO1:FOXO1 ratio (AU)	5.518 ± 2.993	5.930 ± 3.898	0.8164
Autophagy			
*BECLIN1* (MNE)	0.2414 ± 0.0294 (*n* = 6)	0.2749 ± 0.0564	0.2183
*ATG1* (MNE)	0.1709 ± 0.0292	0.1723 ± 0.0246	0.9192
Angiogenesis			
*VEGF* (MNE)	0.9616 ± 0.3412 (*n* = 7)	1.105 ± 0.2710	0.9616
*ANGIOPOIETIN1* (MNE)	0.0211 ± 0.0040 (*n* = 6)	0.0164 ± 0.0033	0.0517
*ANGIOPOIETIN2* (MNE)	0.0662 ± 0.0291	0.0413 ± 0.0098	0.0505
*PAI1* (MNE)	0.0128 ± 0.0051	0.0103 ± 0.0041	0.3428
Cardiac contractility			
p‐PLN: PLN ratio (AU)	0.7646 ± 0.7583	1.128 ± 0.6913 (*n* = 8)	0.3329
SERCA2 (AU)	2.530 ± 0.3793	3.005 ± 1.201 (*n* = 8)	0.3044
Renin–angiotensin system			
*RENIN* (MNE)	0.0004 ± 0.0002 (*n* = 6)	0.0004 ± 0.0005 (*n* = 3)	0.7119
*AT1R* (MNE)	0.0419 ± 0.0351 (*n* = 7)	0.0324 ± 0.0118	0.9452^a^
*AT2R* (MNE)	0.1544 ± 0.0446	0.1202 ± 0.0797	0.3158
*ACE1* (MNE)	0.0101 ± 0.0035	0.0119 ± 0.0049	0.4469
*ACE2* (MNE)	0.0013 ± 0.0007 (*n* = 7)	0.0012 ± 0.0004	0.8060
Oxidative stress			
*SOD1* (MNE)	2.1510 ± 0.3407 (*n* = 6)	1.9240 ± 0.3095	0.2568
*SOD3* (MNE)	0.2940 ± 0.0724 (*n* = 7)	0.2245 ± 0.0461	0.0534
*SOD2* (MNE)	1.929 ± 0.4686	2.976 ± 0.6432	**0.0030**
*CAT* (MNE)	1.0740 ± 0.1375	1.0260 ± 0.1235	0.5050
*eNOS* (MNE)	0.3525 ± 0.1051 (*n* = 6)	0.2877 ± 0.0754	0.2223
*iNOS* (MNE)	0.1517 ± 0.0658	0.1070 ± 0.0292	0.1222
NOX‐2 (AU)	2.438 ± 0.4082	2.378 ± 0.6632 (*n* = 8)	0.8309
Glucose/fatty acid metabolism			
p‐AS160:AS160 ratio (AU)	0.0367 ± 0.0198	0.0623 ± 0.0419 (*n* = 8)	0.1407
PPARγ (AU)	0.0244 ± 0.0118	0.0193 ± 0.0052 (*n* = 8)	0.2810
*PPARγ* (MNE)	0.0612 ± 0.0462 (*n* = 7)	0.0184 ± 0.0045	0.1014^a^

*Note*: *P* < 0.05 was considered statistically significant (shown in bold). Animal numbers used within each subset of the study are shown in brackets. Data were excluded due to technical errors for hormone, and mRNA expression. One outlier was excluded per group using the Grubbs method (α = 0.05), when applicable. Data expressed as means ± SD and were analysed by the unpaired *t*‐test for normally distributed data or Mann–Whitney test for data not normally distributed that indicated by a. Abbreviations: AU, arbitrary unit; MNE, mean normalized expression.

### 3.3 Intrafetal cortisol infusion downregulated markers of cardiac hyperplastic growth, but increased a marker of DNA replication

**FIGURE 2:**
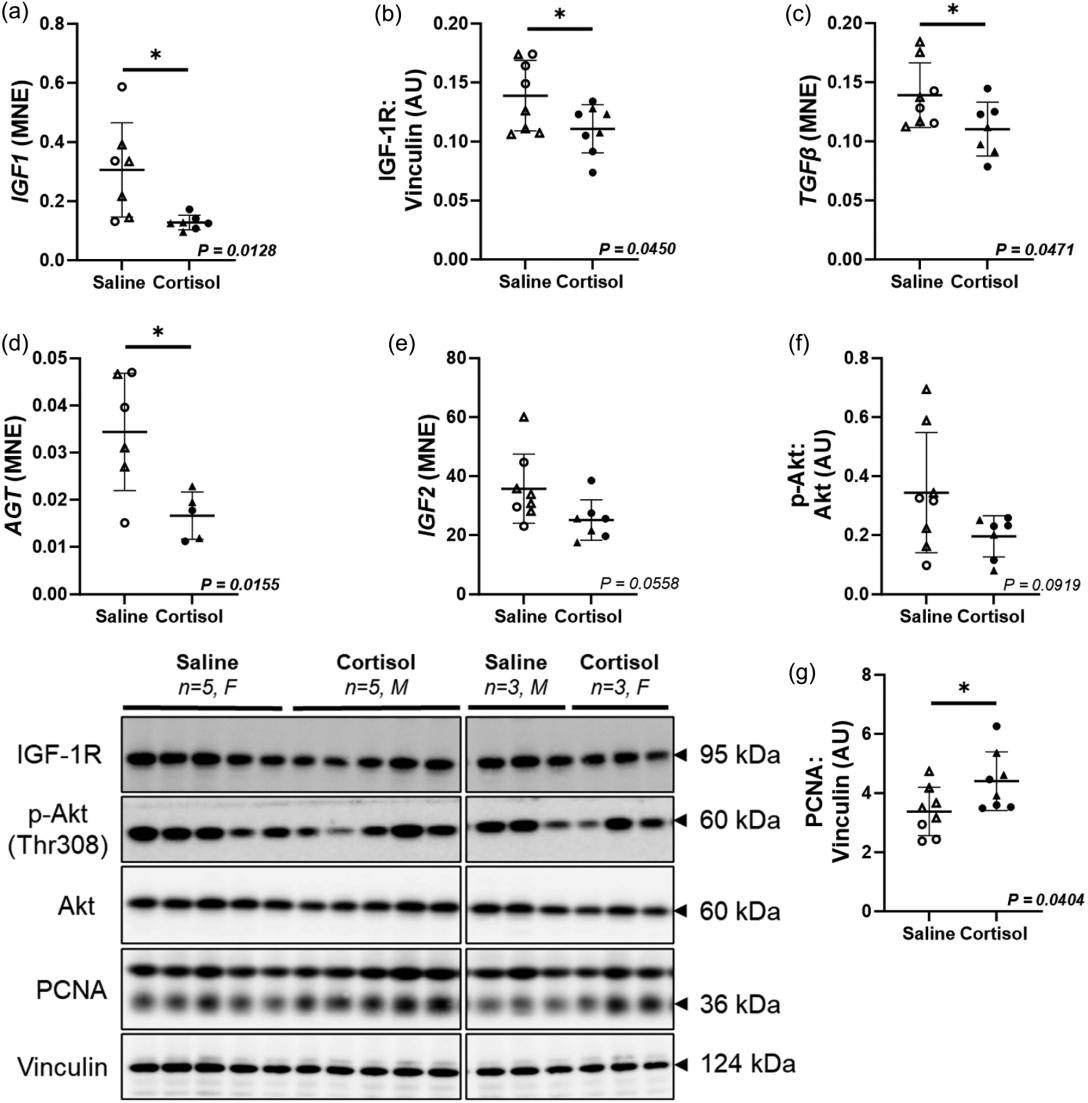
Intrafetal cortisol infusion downregulated markers of cardiac hyperplastic growth, but increased a marker of DNA replication. There was a downregulation in the mRNA or protein expression of *IGF1* (a), IGF‐1R (b), *TGFβ* (c), *AGT* (d), with little decrease in *IGF2* (e) and p‐Akt:Akt ratio (f), as well as an upregulation of PCNA (g) in the Cortisol compared to the Saline group. Males (M), circles; females (F), triangles. Saline, LV tissue from saline‐infused fetuses, open symbols (mRNA: 3M, 5F; protein: 3M, 5F); Cortisol, LV tissue from cortisol‐infused fetuses, filled symbols (mRNA: 4M, 3F; protein: 5M, 3F). mRNA expression was run in triplicate and one sample per animal was run per western blot. Data were excluded due to technical error for mRNA expression. One outlier was excluded per group using the Grubbs method (α = 0.05), when applicable. Data expressed as means ± SD and were analysed by the unpaired *t*‐test. *P *< 0.05 was considered significant. AU, arbitrary unit; LV, left ventricle; MNE, mean normalized expression.

The protein expression of IGF‐1R (*P *= 0.0450) and mRNA of *IGF1* (*P *= 0.0128), *TGFβ* (*P *= 0.0471) and *AGT* (*P *= 0.0155) were downregulated by intrafetal cortisol infusion, but *IGF2* (*P *= 0.0558) and p‐Akt:Akt ratio (*P *= 0.0919; Figure 2a–f) were unchanged. The protein expression of proliferating cell nuclear antigen (PCNA, a marker of DNA replication; *P *= 0.0404; Figure 2g) was upregulated in the Cortisol compared to the Saline group. There was no change in the mRNA or protein expression of other markers involved in cardiac growth (*IGFBP2*, *IGF1R*, *IGF2R*, *RPS6KB1*, *ANP*, *PCNA*, *P21*, *P27*, *CYCLIN D1*, *GATA4*, *GATA6*, *CDC2*, *CHEK*1, *C‐KIT*, *CD31*, p‐mTOR:m‐TOR ratio, p‐P70 S6K:P70 S6K ratio), apoptosis (*BAX*, *BCL2*, *c‐MYC*, *BIRC5*, p‐FOXO1:FOXO1 ratio), autophagy (*BECLIN1*, *ATG1*), angiogenesis (*VEGF*, *ANGIOPOIETIN1*, *ANGIOPOIETIN2*, *PAI1*), cardiac contractility (p‐PLN:PLN ratio, SERCA‐2), and renin–angiotensin system (*RENIN*, *AT1R*, *AT2R*, *ACE1*, *ACE2*), between Saline and Cortisol groups (Table 2).

### 3.4 Intrafetal cortisol infusion upregulated complex I of the electron transport chain and disrupted the relationship between OXPHOS complexes and GR isoforms

**FIGURE 3:**
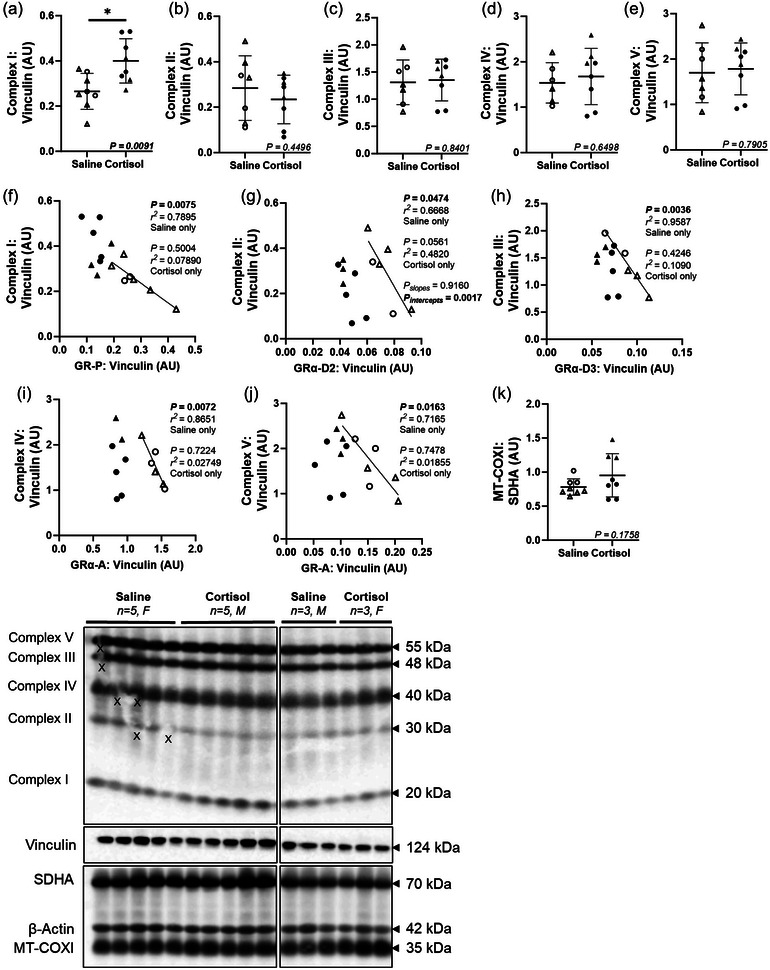
Intrafetal cortisol infusion upregulated complex I of the electron transport chain and disrupted the normal relationship between OXPHOS complexes and GR isoforms. The protein expression of complex I (a) was upregulated by cortisol infusion, while there were no changes in the complex II–V (b–e). In the Saline only, there were negative linear relationships with OXPHOS complexes and GR isoforms (f–j). There was no change in the marker of mitochondrial biogenesis (MT‐COX1:SDHA ratio; k), between Saline and Cortisol groups. Males (M), circles; females (F), triangles. Saline, LV tissue from saline‐infused fetuses, open symbols (protein: 3M, 5F); Cortisol, LV tissue from cortisol‐infused fetuses, filled symbols (protein: 5M, 3F). One sample per animal was run per western blot. One outlier was excluded per group using the Grubbs method (α = 0.05), when applicable. Data expressed as means ± SD and were analysed by the unpaired *t*‐test. To assess the relationship between two measures, simple linear regression was used. *P *< 0.05 was considered significant. X indicates data excluded from analysis (due to artifacts affecting the bands). AU, arbitrary unit; LV, left ventricle.

The protein expression of complex I (*P *= 0.0091; Figure 3a) and mRNA expression of *SOD2* (*P *= 0.0030; Table 2) were upregulated by cortisol infusion within the fetal heart, whereas there was no change in the other OXPHOS complexes (II–V; Figure 3b–e) or mitochondrial biogenesis (determined by a ratio of MT‐COX1:SDHA; Figure 3k) between groups. In the Saline but not the Cortisol group, there was a negative relationship between specific GR subunits and OXPHOS complexes, including GRα‐A and complex I (*P *= 0.0283, *R* ^2^ = 0.5793; Supporting information, Figure S1), complex III (*P *= 0.0195, *R* ^2^ = 0.6963; Figure S1), complex IV (*P *= 0.0072, *R* ^2^ = 0.8651; Figure 3i) and complex V (*P *= 0.0112, *R* ^2^ = 0.7546; Figure S1); GR‐P and complex I (*P *= 0.0075, *R* ^2^ = 0.7895; Figure 3f) and complex V (*P *= 0.0395, *R* ^2^ = 0.6938; Figure S1); GR‐A and complex I (*P *= 0.0409, *R* ^2^ = 0.5288; Figure S1) and complex V (*P *= 0.0163, *R* ^2^ = 0.7165; Figure 3j); and GRα‐D3 and complex III (*P *= 0.0036, *R* ^2^ = 0.9587; Figure 3h), complex IV (*P *= 0.0126, *R* ^2^ = 0.9750; Figure S1) and complex V (*P *= 0.0226, *R* ^2^ = 0.8625; Figure S1). Interestingly, there were also negative relationships between GRα‐D2 and several OXPHOS complexes in either Saline or Cortisol only when data were stratified by treatment such as GRα‐D2 with complex I (Saline only: *P *= 0.0162, *R* ^2^ = 0.7173; Figure S1), complex II (Saline only: *P *= 0.0474, *R* ^2^ = 0.6668; Figure 3g), complex III (Saline: *P *= 0.0039, *R* ^2^ = 0.9001; Cortisol: *P *= 0.0432, *R* ^2^ = 0.5212; Figure S1), complex IV (Cortisol only: *P *= 0.0299, *R* ^2^ = 0.5718; Figure S1) and complex V (Saline: *P *= 0.0019, *R* ^2^ = 0.9291; Cortisol: *P *= 0.0377, *R* ^2^ = 0.5404; Figure S1). Gene and protein expression of markers of oxidative stress (*SOD1*, *SOD3*, *CAT*, *eNOS*, *iNOS* and NOX‐2) were not different between treatment groups (Table 2).

### 3.5 Intrafetal cortisol infusion upregulated markers of insulin‐dependent cardiac glucose uptake

**FIGURE 4:**
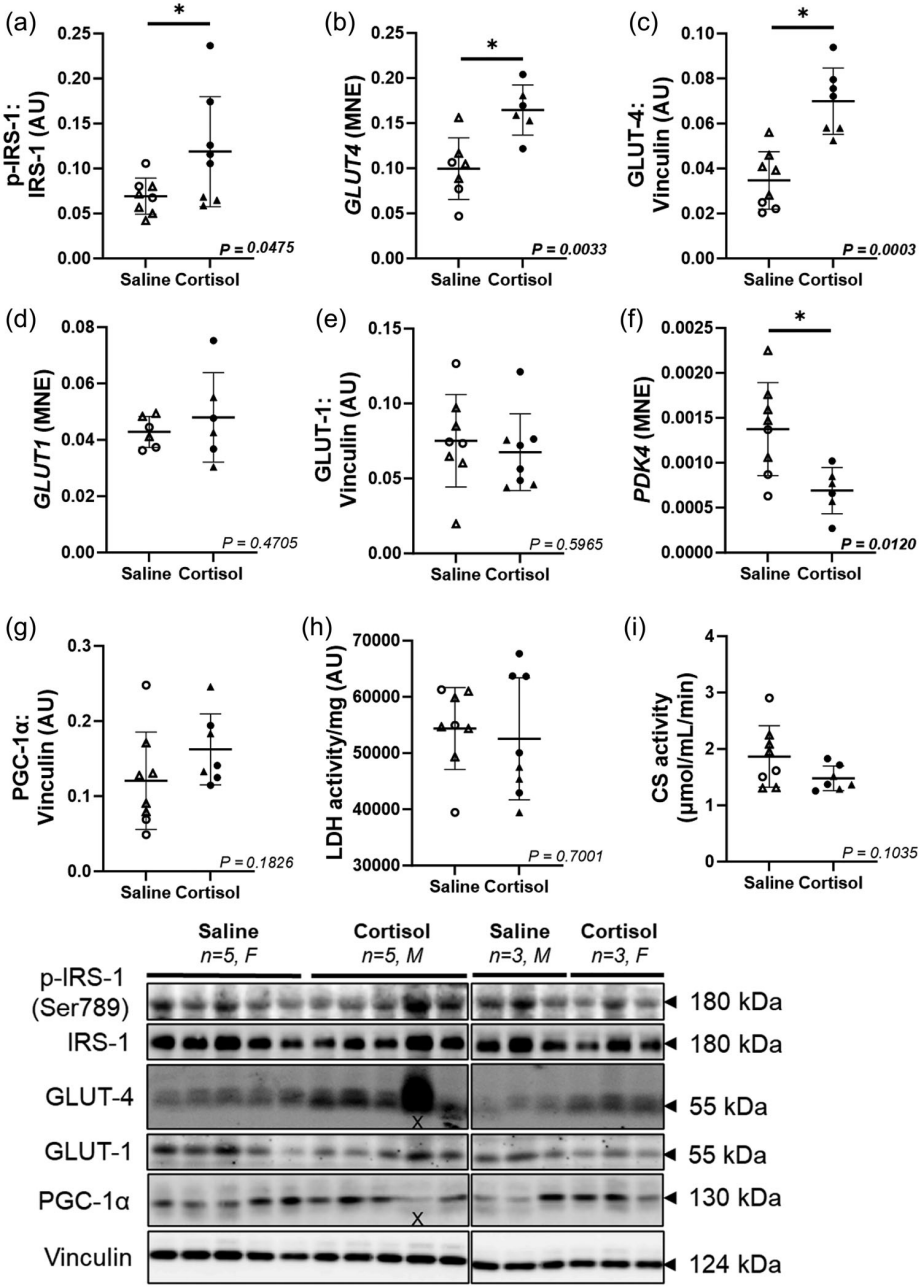
Intrafetal cortisol infusion upregulated markers of insulin‐dependent cardiac glucose uptake. There was upregulation in the protein expression of p‐IRS‐1:IRS‐1 ratio (a), and gene and protein expression of GLTU‐4 (b, gene; c, protein) in the cortisol‐infused heart, while GLUT‐1 (d, gene; e, protein) remained unchanged. The mRNA expression of *PDK4* (f) was downregulated by cortisol infusion. The protein abundance of PGC‐1α (g), and enzyme activity of LDH (h), and CS (i) were not different between Saline and Cortisol groups. Males (M), circles; females (F), triangles. Saline, LV tissue from saline‐infused fetuses, open symbols (mRNA: 3M, 5F; protein/enzymes: 3M, 5F); Cortisol, LV tissue from cortisol‐infused fetuses, filled symbols (mRNA: 4M, 3F; protein/enzymes: 5M, 3F). mRNA expression was run in triplicate and one sample per animal was run per western blot. Data were excluded due to technical error for mRNA expression. One outlier was excluded per group using the Grubbs method (α = 0.05), when applicable. Data expressed as means ± SD and were analysed by the unpaired *t*‐test. *P *< 0.05 was considered significant. (X) indicates data excluded from analysis (due to artifacts affecting the bands). AU, arbitrary unit; CS, citrate synthase; LDH, lactate dehydrogenase; LV, left ventricle; MNE, mean normalized expression.

Cortisol infusion increased the cardiac expression of phosphorylated IRS‐1 (*P *= 0.0475; Figure 4a) and both mRNA and protein expression of insulin‐dependent GLUT‐4 (*P *= 0.0033, *P *= 0.0003; Figure 4b,c). There was no difference in mRNA or protein expression of GLUT‐1 (Figure 4d,e) between treatment groups. The mRNA expression of *PDK4* (*P *= 0.0120, Figure 4f) was downregulated by intrafetal cortisol infusion. Neither protein and gene expression of peroxisome proliferator‐activated receptor γ coactivator 1‐α (PGC‐1α) nor enzyme activity of LDH and CS were different between treatment groups (Figure 4g–i). There was no change in the protein or gene expression of p‐AS160:AS160 ratio and peroxisome proliferator‐activated receptor γ (PPARγ) between treatment groups (Table 2).

### 3.6 Intrafetal cortisol infusion downregulated signalling molecules in cardiovascular protection

**FIGURE 5:**
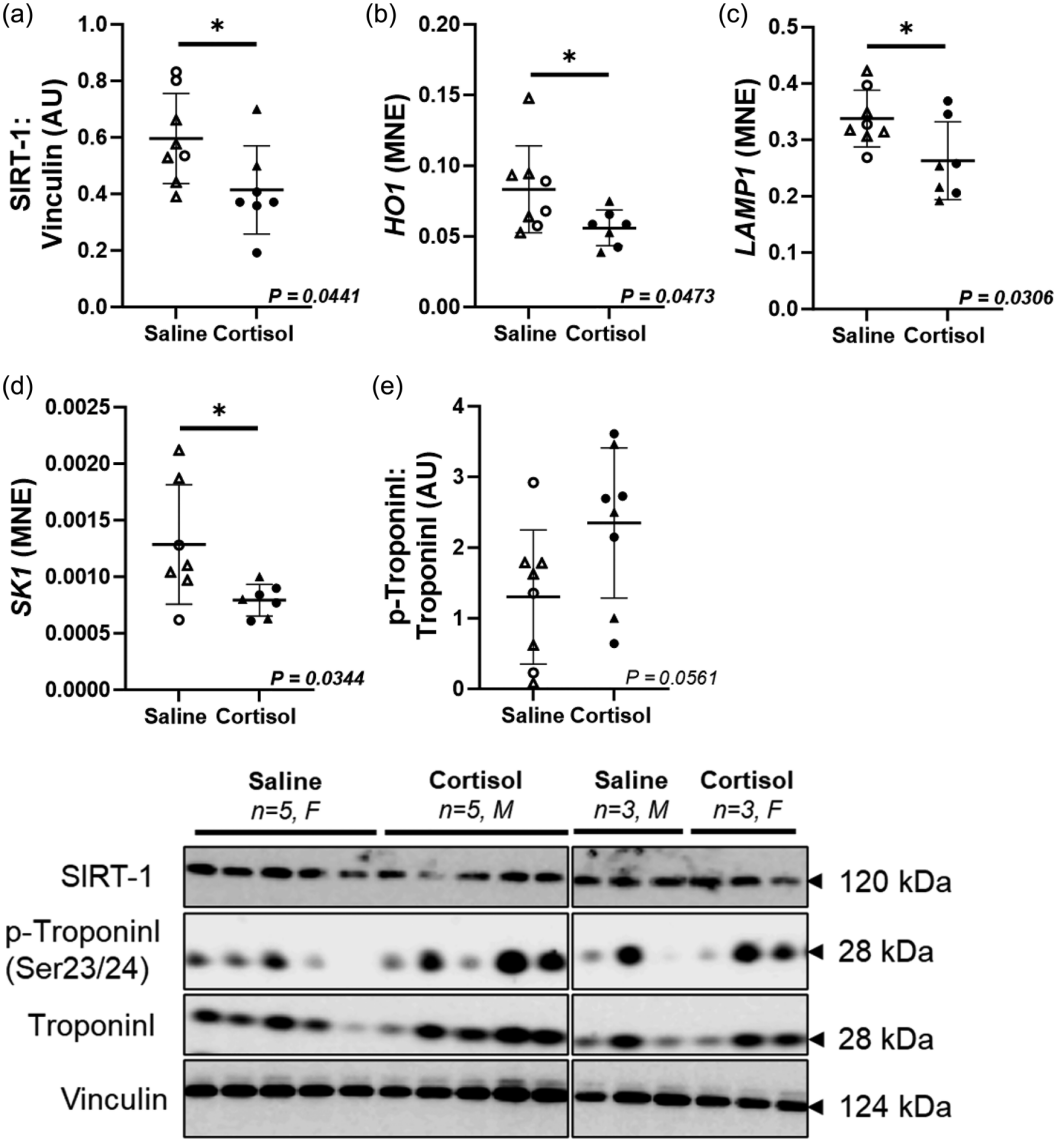
Intrafetal cortisol infusion downregulated signalling molecules in cardiovascular protection. The mRNA or protein expression of SIRT‐1 (a), *HO1* (b), *LAMP1* (c) and *SK1* (d) was downregulated by intrafetal cortisol infusion. The cardiac expression of phosphorylated troponin I (e) had little increase in the Cortisol group. Males (M), circles; females (F), triangles. Saline, LV tissue from saline‐infused fetuses, open symbols (mRNA: 3M, 5F; protein/enzymes: 3M, 5F); Cortisol, LV tissue from cortisol‐infused fetuses, filled symbols (mRNA: 4M, 3F; protein/enzymes: 5M, 3F). mRNA expression was run in triplicate and one sample per animal was run per western blot. Data were excluded due to technical error for mRNA expression. One outlier was excluded per group using the Grubbs method (α = 0.05), when applicable. Data expressed as means ± SD and were analysed by the unpaired *t*‐test. *P *< 0.05 was considered significant. AU, arbitrary unit; LV, left ventricle; MNE, mean normalized expression.

The cardiac protein expression of SIRT‐1 (*P *= 0.0441) and mRNA expression of *HO1* (*P *= 0.0473), *LAMP1* (*P *= 0.0306) and *SK1* (*P *= 0.0344) were downregulated in the Cortisol compared to the Saline group (Figure 5a–d). The cardiac expression of phosphorylated troponin I (*P *= 0.0561; Figure 5e) was not different between treatment groups.

### 3.7 Intrafetal cortisol infusion did not alter glycogen and collagen deposition, nor Ki67 staining

**FIGURE 6:**
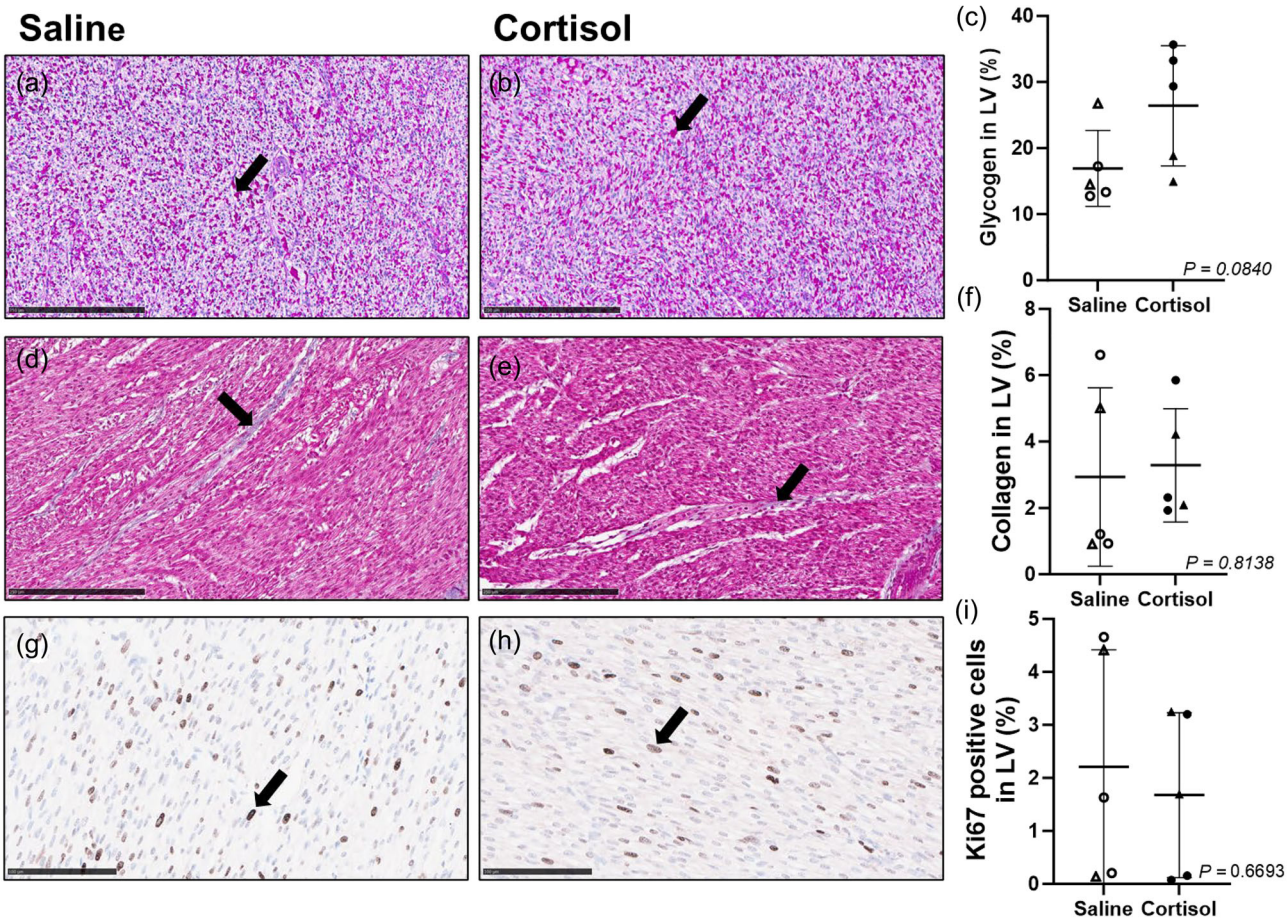
Intrafetal cortisol infusion did not alter glycogen and collagen deposition, nor Ki67 staining. (a, b) ×20 magnification representative micrograph of glycogen staining by PAS (black arrow) in Saline (a) and Cortisol (b). (d, e) ×20 magnification representative micrograph of collagen staining by Masson's trichrome (black arrow) in Saline (d) and Cortisol (e). (g, h) ×40 magnification representative micrograph of Ki67 staining by IHC (black arrow) in Saline (g) and Cortisol (h). (c, f) There was no difference in the glycogen (c) and collagen (f) deposition between the Saline and Cortisol groups. There was also no difference in the Ki67 staining between the treatment groups (I). Males (M), circles; females (F), triangles. Saline, LV tissue from saline‐infused fetuses, open symbols (glycogen/collagen staining: 3M, 2F; Ki67 staining: 2M, 1F); Cortisol, LV tissue from cortisol‐infused fetuses, filled symbols (glycogen/collagen staining: 3M, 2F; Ki67 staining: 1M, 2F). A smaller subset of animals were included in this analysis due to missing fixed tissue samples (Saline, *n* = 3, Cortisol, *n* = 3). Scale bars of 250 µm (glycogen/collagen staining) and 100 µm (Ki67 IHC staining). Data expressed as means ± SD and were analysed by the unpaired *t*‐test. *P *< 0.05 was considered significant. LV, left ventricle.

There was no change in the glycogen and collagen deposition of fetal LV between the Saline and Cortisol groups (Figure 6c,f). There was also no difference in Ki67 IHC staining of fetal LV between treatment groups (Figure 6i).

## 4 DISCUSSION

Cortisol is a hormone that plays a key role in the differentiation and maturation of many organs, including the lung, liver and kidney, in preparation for birth (Fowden et al., 2016; Giraud et al., 2006). However, its role in cardiac development remains less clear. Five previous studies have investigated the role of intrafetal cortisol infusion on cardiac development in sheep (Fahmi et al., 2004; Giraud et al., 2006; Lumbers et al., 2005; Rudolph et al., 1999; Segar et al., 1995); however, the results suggest an acceleration, delay or no change in cardiac maturation due to variations in gestational age, duration and dose of cortisol administration. Our data indicate that elevated cortisol concentrations in preterm fetuses altered molecular markers involved in cardiac glucocorticoid signalling, proliferation, metabolism and cardioprotection, driving early cardiac maturation and potentially compromising long‐term cardiovascular health.

The aim of this study was to increase fetal plasma cortisol concentrations to those observed in late gestation after the prepartum surge in cortisol but in a preterm fetus. We showed that a 7‐day infusion of cortisol to preterm fetuses (109–116 dG) led to a rise in fetal plasma and cardiac cortisol concentrations. Although the fetal plasma cortisol concentration (15.62 ng/mL) is lower than those reported in the previous studies (Giraud et al., 2006; Lumbers et al., 2005), it is more physiologically relevant to those seen in late gestation. In sheep, the prepartum surge in fetal cortisol concentrations begins around 130–134 dG (∼10–20 ng/mL), peaking around birth (∼90–110 ng/mL, ∼150 dG). Prior to this, between 100 and 125 dG (mid‐late gestation), fetal plasma cortisol concentrations are below 1 ng/mL (Dimasi et al., 2023; Magyar et al., 1980; Phillips et al., 1996). Although heart weight was not different between Cortisol and Saline groups, the mRNA or protein expression of cardiac hyperplastic growth markers (*IGF1*, IGF‐1R, *TGFβ* and *AGT*) was reduced in fetuses that received a cortisol infusion. Consistent with our findings, Rudolph et al. (1999) also found that cortisol infusion during late gestation (131–135 dG) inhibited cardiomyocyte proliferation in fetal sheep. They also observed fetal plasma cortisol concentrations (18.1 ng/mL) comparable to those in our study (Rudolph et al., 1999). Insulin‐like growth factor 1 (IGF‐1) stimulates the proliferation of cardiomyocytes in culture (Sundgren et al., 2003), and high concentrations of cortisol at term are associated with the downregulation of IGF‐1 and IGF‐2 in skeletal muscle in fetal sheep (Li et al., 2002) as well as fetal cardiac expression of *IGF1R* mRNA (Reini et al., 2006). However, Giraud et al. (2006) showed that cortisol infusion increased plasma cortisol concentration without changing blood pressure and increased cardiac proliferative growth with no change in hypertrophic growth. On the other hand, Lumbers et al. (2005) showed that a high dose of cortisol infusion contributed to cardiac growth via hypertrophy rather than proliferation, but was also associated with severe hypertension, which itself is known to increase hypertrophic growth of the heart (Giraud et al., 2006; Louey & Thornburg, 2005). In this study, we found that cortisol infusion upregulated the protein expression of PCNA, a marker of DNA replication, which could either contribute to proliferation within the heart or increase cardiac binucleation as no other markers of proliferation were upregulated. However, Giraud et al. (2006) observed an increase in Ki67 staining (as a marker of DNA duplication) in both LV and right ventricle (128–135 dG, 7 days), whereas in our study. There was no difference in Ki67 staining of the fetal LV between the Saline and Cortisol groups. Timing in gestation and duration of intervention, fetal plasma cortisol concentration, and expression profiles of GR isoforms are possible contributing factors that may explain differences observed in cardiac hyperplastic growth.

Glucocorticoids act via the GR to play a significant role in cardiovascular health and disease (Liu et al., 2019), with a critical role of GR in cardiac development (Kim et al., 2014; Rog‐Zielinska et al., 2015, 2013). The gene encoding GR is nuclear receptor subfamily 3, group C, member 1 (*NR3C1*), which is translated to various isoforms of GR protein via alternative splicing or alternative translation initiation (Lockett et al., 2024). Multiple GR isoforms have been identified in the placenta and lung of humans (Saif et al., 2015) and sheep (Clifton et al., 2019; Orgeig et al., 2015), and their abundance varies with gestational age, fetal sex, glucocorticoid exposure and pregnancy complications (Clifton et al., 2019; Saif et al., 2014). Interestingly, we found that protein expression of five GR isoforms (GRα‐A, GR‐P, GR‐A, GRα‐D2 and GRα‐D3) was downregulated by intrafetal cortisol infusion, which may explain the alterations in the markers of cardiac hyperplastic growth and metabolism. In line with this, Orgeig et al. (2015) also found that experimental placental restriction (through carunclectomy) in fetal sheep induced intrauterine growth restriction (IUGR), resulting in elevated plasma cortisol concentrations and reduced GRα‐A expression in the fetal lungs. Generally, following glucocorticoid binding, the GR activates or suppresses the transcription of target genes, potentially affecting 10–20% of the human genome, and post‐translational modification of GR isoforms further expands the diversity in gene‐regulatory and functional profiles (Oakley & Cidlowski, 2013). Information on the physiological function and signalling pathway of each GR isoform is scarce. GRα‐A is the main functional isoform and regulates multiple genes, including those involved in cardiac development, via interaction with glucocorticoid response elements (GREs) (Lu & Cidlowski, 2005). GR‐P and GR‐A contribute to glucocorticoid resistance (Lu & Cidlowski, 2004). In contrast to the GRα‐A, which localizes in the cytoplasm of cells in the absence of hormone and translocates to the nucleus upon glucocorticoid binding, the GRα‐D isoform resides constitutively in the nuclei of cells (Oakley & Cidlowski, 2013). Additionally, nuclear‐localized GRα‐D associates with glucocorticoid response element (GRE)‐containing promoters of specific target genes independent of glucocorticoid treatment (Lu et al., 2007; Oakley & Cidlowski, 2013). In addition to the observed downregulation of GR isoforms, other glucocorticoids including cortisone, 11‐deoxycortisol and corticosterone were lower in the cortisol‐infused hearts. This could imply that cortisol binds to tissue GRs and exerts negative feedback on the hypothalamic–pituitary–adrenal axis, suppressing the production of endogenous glucocorticoids (Almanza‐Sepulveda et al., 2020; Lu & Cidlowski, 2006).

The effects of glucocorticoids on energy uptake and metabolism in the adult heart have been well‐defined (Davies et al., 2022); however, the role of cortisol on mitochondrial OXPHOS in the preterm fetal heart remains largely unknown. In adult cardiomyocytes, energy in the form of ATP is generated through mitochondrial OXPHOS (Zhao et al., 2019). In this study, cortisol infusion had no impact on mitochondrial abundance or CS activity; however, protein abundance of OXPHOS complex I, the entry point of the electron transport chain, was increased by cortisol infusion. Given that complex I is a major site for superoxide generation in mitochondria (Johnson Jr et al., 2003; Kudin et al., 2004; Kushnareva et al., 2002; Lenaz et al., 2002; Liu et al., 2002), an increase in complex I may suggest early developmental programming of mitochondrial dysfunction in response to cortisol infusion. In line with this finding, the mRNA expression of *SOD2*, a mitochondrial antioxidant marker, was upregulated by cortisol infusion. This may be in response to the oxidative stress created at the mitochondrial matrix due to cortisol‐induced increases in complex I. Importantly, we observed negative relationships between OXPHOS complexes and several GR isoforms, but only in the Saline group. This suggests that in normal fetuses, GR may be a driver of OXPHOS complexes, but excess cortisol in mid‐late gestation dysregulates this relationship. However, we found negative relationships with GRα‐D2 and complexes III and V in both the Saline and Cortisol groups.

In mammals, the fetal heart primarily utilizes glucose and lactate to produce ATP, whereby glucose is transported into cardiomyocytes mainly via two solute carriers: GLUT‐1 and GLUT‐4 (Devaskar & Mueckler, 1992; Fisher et al., 1980). GLUT‐4 is the predominant GLUT expressed in the adult heart, while GLUT‐1 is the major transporter in fetal cardiomyocytes (Smoak & Branch, 2000). Cortisol increases the storage of glucose in a variety of fetal tissues (Fowden et al., 1996). Interestingly, we found that both gene and protein expression of GLUT‐4 were upregulated due to cortisol infusion, while GLUT‐1 remained unchanged. In comparison, Jellyman et al. (2012) reported that GLUT‐4 protein abundance in skeletal muscle in the sheep fetus was increased by maternal treatment with dexamethasone, a synthetic glucocorticoid, without any effect on the components of insulin signalling pathway such as p‐Akt and protein kinase C (Jellyman et al., 2012). GLUT‐4 protein increased in the LV of chronically anaemic sheep fetuses while GLUT‐1 decreased, pointing to the importance of GLUT‐4 in the stressed fetal heart (Ralphe et al., 2005). Other stressors, such as hypoxia and ischaemia, trigger the translocation of the insulin‐responsive GLUT‐4 to the plasma membrane of cardiomyocytes in adults (Slot et al., 1991; Sun et al., 1994). AS160 plays a major role in this translocation, and it is phosphorylated by insulin receptor substrate‐1 (IRS‐1) (Hay Jr, 1994; Jewell et al., 2016; Taniguchi et al., 2006). Interestingly, we found that cardiac expression of phosphorylated IRS‐1 was increased in the Cortisol group, providing evidence that 7‐day cortisol infusion into the preterm fetus may enhance cardiac glucose uptake through activation of the insulin signalling pathway and upregulation of GLUT‐4 expression. This may suggest the onset of an early cardiac maturation process. In addition, mRNA expression of pyruvate dehydrogenase kinase 4 (*PDK4*), which mediates the metabolic switch from glucose to fatty acids (Rowles et al., 1996), was downregulated by cortisol, indicating more reliance on glucose as a cardiac fuel and may, in part, explain the increase in GLUT‐4.

SIRT‐1 is recognized for its cardioprotective role, exerting anti‐apoptotic effects, regulating oxidative stress, modulating metabolic pathways, inhibiting inflammation and promoting cardiomyocyte proliferation (Cheng et al., 2003; Ma & Li, 2015; McBurney et al., 2003; Wu et al., 2022). Cardiac protein expression of SIRT‐1 was downregulated as a result of elevated cortisol concentrations in this study. This may further support our findings of reduced GR and cardiac hyperplastic growth markers, indicating early maturation in the developing heart. In addition, we found that other genes involved in cardiovascular protection, including *HO1*, *LAMP1* and *SK1*, were also downregulated due to intrafetal cortisol infusion. Haem oxygenase‐1 (HO1) is a stress response protein (Wu et al., 2011), and its knockdown in mice leads to exaggerated cardiac injury and dysfunction after ischaemia–reperfusion compared with wild‐type hearts (Yoshida et al., 2001). Lysosomal‐associated membrane protein 1 (LAMP1) plays a crucial role in autophagy, a process that is activated in pathological conditions such as heart failure (Hesketh et al., 2010) and the diabetic heart (Xie et al., 2011). The activity of sphingosine kinase 1 (SK1) leads to the generation of sphingosine 1‐phosphate (S1P) in the heart, and the application of S1P to cultured cardiomyocytes under hypoxic conditions or treatment of isolated hearts either before ischaemia or at the beginning of reperfusion exerts beneficial effects on cell survival (Karliner, 2009). Taken together, these findings suggest that an early increase in fetal cortisol concentrations, occurring before the typical prepartum rise, may have two key effects: (1) accelerating cardiac maturation and (2) compromising cardioprotective pathways – which together may predispose the offspring to a higher risk of CVDs after birth unless cardiac growth is normalized before birth.

Molecular alterations in cardiometabolic pathways occur naturally during fetal heart maturation near‐term, some of which may align with the findings of this study. We have previously shown that in near‐term fetuses (140 dG), the cortisol: cortisone ratio was higher, while other glucocorticoids such as 11‐deoxycortisol and corticosterone were lower compared to preterm (116 dG) hearts (Amanollahi et al., 2025). This hormonal profile closely resembles the changes induced by cortisol infusion in this study. However, a strong positive correlation between cortisol and T_3_ concentrations was observed exclusively in near‐term fetuses (Amanollahi et al., 2025), a relationship that was absent in cortisol‐infused fetuses. The cardiac protein expression of GR isoforms and IGF‐1R decreased, while PCNA increased from preterm to near‐term (Amanollahi et al., 2025), a pattern similar to that observed in the current study following cortisol infusion. Moreover, IRS‐1 phosphorylation and GLUT‐4 expression also increased toward term (Amanollahi et al., 2025), a phenomenon similarly observed in cortisol‐infused fetuses. Overall, intrafetal cortisol infusion in preterm fetuses induced molecular alterations in GR signalling, cardiac growth, and metabolism, resembling natural maturation near term. While these changes may have long‐term consequences if sustained into adulthood, they could also serve as adaptive responses to fetal stress, particularly in cases of preterm birth.

There are some limitations to this study that may be worth exploring in the future. High‐resolution respirometry of mitochondrial OXPHOS activity and reactive oxygen species production were not performed due to the requirement for fresh tissue samples. The LV tissues that we used in the current study were generated as part of a programme of work designed with the primary objective of investigating fetal adrenal physiology (Adams et al., 1999; Ross et al., 2000). Therefore, cardiac function and blood pressure were not measured and remain an important avenue to investigate in future studies as cortisol can cause vasoconstriction and increased afterload, which also impact cardiac molecular signalling (Giraud et al., 2006). An additional limitation of the work is the sample size, which prevented us from interrogating sex differences in the expression of cardiac GR isoforms and their response to chronic exposure of exogenous glucocorticoids (Saif et al., 2015). The fetal plasma cortisol concentrations observed in this study were within the physiological range in late gestation but in a preterm fetus, at a time when cardiomyocytes just begin to transition from mononucleated and proliferative to binucleated and hypertrophic. Further studies are needed to investigate the dose‐dependent impacts of cortisol at different stages of cardiac maturation to better define the threshold at which significant cardiac changes occur.

## 5 CONCLUSION

This study provides new insights into the effects of chronic cortisol exposure in preterm fetuses, revealing changes in key molecular pathways involved in cardiac glucocorticoid signalling, proliferation, metabolism and cardioprotection. Cortisol infusion downregulated GR isoforms, reduced proliferation markers, and increased DNA replication markers, suggesting an accelerated shift toward cardiac maturation. The concurrent reduction in GR expression and elevated cortisol concentrations may indicate glucocorticoid desensitization, potentially disrupting metabolic homeostasis. Upregulation of insulin‐dependent glucose markers further supports early maturation, though it may also reflect a compensatory adaptation to limited fatty acid and oxygen availability in the preterm heart. Additionally, the downregulation of cardioprotective markers suggests increased vulnerability to future cardiac injury. Overall, while cortisol‐induced maturation may benefit a stressed fetus, particularly if born early, it may also predispose the heart to long‐term cardiovascular risks. Longitudinal studies are needed to determine whether these molecular changes contribute to programmed CVD.

## AUTHOR CONTRIBUTIONS

Conception or design of the work: I. Caroline McMillen, Janna L. Morrison. Acquisition or analysis or interpretation of data for the work: Reza Amanollahi, Stacey L. Holman, Melanie Rose Bertossa, Ashley S. Meakin, Vicki L. Clifton, Kent L. Thornburg, I. Caroline McMillen, Michael D. Wiese, Mitchell C. Lock, Janna L. Morrison. Drafting the work or revising it critically for important intellectual content: Reza Amanollahi, Stacey L. Holman, Melanie Rose Bertossa, Ashley S. Meakin, Kent L. Thornburg, Michael D. Wiese, Mitchell C. Lock, Janna L. Morrison. All authors have read and approved the final version of this manuscript and agree to be accountable for all aspects of the work in ensuring that questions related to the accuracy or integrity of any part of the work are appropriately investigated and resolved. All persons designated as authors qualify for authorship, and all those who qualify for authorship are listed.

## CONFLICT OF INTEREST

None declared.

## Supporting information



Table S1. List of antibodies used for cardiac protein expression analysis via western blot.
Figure S1. Intrafetal cortisol infusion disrupted normal relationship between OXPHOS complexes and GR isoforms.

## Data Availability

All data supporting the results are presented in the manuscript.
