# Supplementary material for: Elevated cortisol concentration in preterm sheep fetuses impacts heart development
Source: Exp Physiol. 2025 Apr 28;110(12):1928–47. doi: 10.1113/EP092506 (PMC12665941; doi:10.1113/EP092506)
Supplement: Supplementary file 1 — Table S1. List of antibodies used for cardiac protein expression analysis via western blot. Figure S1. Intrafetal cortisol infusion disrupted normal relationship between OXPHOS complexes and GR isoforms. [file EPH-110-1928-s001.docx]

**Table S1.** List of antibodies used for cardiac protein expression analysis via western blot.

| **Antibody** | **Concentration** | **Catalogue #** | **Company** |
| --- | --- | --- | --- |
| Total OXPHOS | 1:500 | ab110413 | Abcam |
| MitoBiogenesis | 1:250 | ab123545 | Abcam |
| PGC-1α | 1:1000 | 2178S | Cell Signaling Technology |
| GLUT-4 | 1:1000 | ab33780 | Abcam |
| GLUT-1 | 1:1000 | sc-7903 | Santa Cruz Biotechnology |
| SERCA2 | 1:1000 | ab137020 | Abcam |
| SIRT-1 | 1:1000 | 9475 | Cell Signaling Technology |
| Troponin I | 1:1000 | 4002 | Cell Signaling Technology |
| p-Troponin I(Ser23/24) | 1:1000 | 4004S | Cell Signaling Technology |
| AS160 | 1:1000 | 2670S | Cell Signaling Technology |
| p-AS160(Thr642) | 1:1000 | 4288S | Cell Signaling Technology |
| Phospholamban (PLN) | 1:1000 | 8495 | Cell Signaling Technology |
| p-PLN(Ser16/Thr17) | 1:1000 | 8496S | Cell Signaling Technology |
| Akt | 1:1000 | 9272S | Cell Signaling Technology |
| p-Akt(Thr308) | 1:1000 | 9275S | Cell Signaling Technology |
| IRS-1 | 1:1000 | 3194 | Cell Signaling Technology |
| p-IRS-1(Ser789) | 1:1000 | 2389S | Cell Signaling Technology |
| Total GR | 1:1000 | A303-491A | Bethyl Laboratories |
| mTOR | 1:1000 | 2972S | Cell Signaling Technology |
| p-mTOR(Ser2448) | 1:1000 | 2971S | Cell Signaling Technology |
| IGF-1R | 1:1000 | 3027S | Cell Signaling Technology |
| P70 S6 Kinase | 1:1000 | 9202 | Cell Signaling Technology |
| p-P70 S6 Kinase(Thr389) | 1:1000 | 9205 | Cell Signaling Technology |
| FOXO1 | 1:1000 | 9454 | Cell Signaling Technology |
| p-FOXO1(Thr24) | 1:1000 | 9464 | Cell Signaling Technology |
| PPARγ | 1:1000 | 2435 | Cell Signaling Technology |
| NOX-2 | 1:1000 | ab129068 | Abcam |
| PCNA | 1:2000 | 2586 | Cell Signaling Technology |
| Vinculin | 1:2000 | 18799S | Cell Signaling Technology |


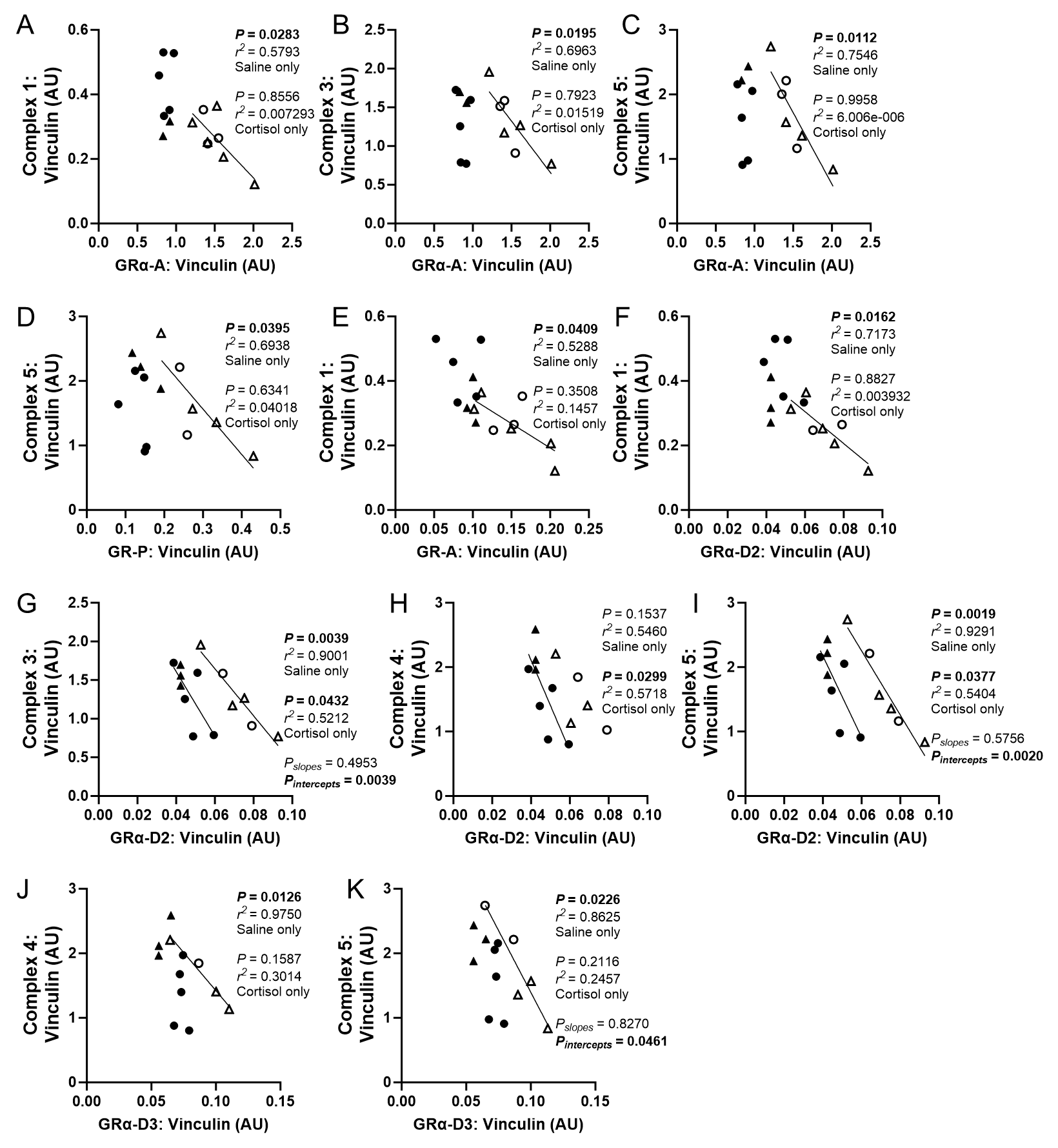


**Figure S1. Intrafetal cortisol infusion disrupted normal relationship between OXPHOS complexes and GR isoforms.** There were negative linear relationship between GRα-A and complex 1 (A), complex 3 (B), and complex 5 (C) in Saline only. There were negative linear relationship between GR-P and complex 5 (D) in Saline only. There were negative linear relationship between GR-A and complex 1 (E) in Saline only. There were negative linear relationship between GRα-D2 and complex 1 (F), complex 3 (G), complex 4 (H), and complex 5 (I) in Saline and/or Cortisol. There were negative linear relationship between GRα-D3 and complex 4 (J), and complex 5 (K) in Saline only. Males (M)=circles, females (F)=triangles. Saline, left ventricle (LV) tissue from saline-infused fetuses, open symbols (protein=3M, 5F). Cortisol, LV tissue from cortisol-infused fetuses, filled symbols (protein=5M, 3F). mRNA expression was run in triplicate and one sample per animal was run per western blot. To assess the relationship between two measures, simple linear regression was used. Data expressed as mean ± SD. *P*<0.05 was considered significant. AU: arbitrary unit.
